# Editorial: Plant responses to phosphorus and nitrogen starvation: genetic insights and agricultural innovations

**DOI:** 10.3389/fpls.2025.1772847

**Published:** 2026-01-20

**Authors:** Viswanathan Satheesh, Donald James

**Affiliations:** 1 Genome Informatics Facility, Office of Biotechnology, Iowa State University, Ames, IA, United States; 2 Forest Biotechnology Department, KSCSTE-Kerala Forest Research Institute, Thrissur, Kerala, India

**Keywords:** adaptive mechanisms, adaptive response, agricultural soils, agronomic nitrogen use efficiency, agronomic NUE, ammonium transporters, arabidopsis, below-ground foraging

## Introduction

Nitrogen and phosphorus are the two critical macronutrients limiting agricultural productivity worldwide. As global agriculture strives to meet the food demands of a rapidly increasing population while reducing ecological impact, understanding how plants perceive, respond to, and adapt to nutrient deficiencies is crucial to ensure sustainable agriculture. The articles compiled in this Research Topic represent a significant step forward in deciphering the intricate molecular networks that govern plant responses to nitrogen and phosphate starvation, offering both fundamental insights and practical pathways toward developing nutrient-efficient crop varieties that promote sustainable agriculture. Plants have evolved sophisticated sensory and regulatory systems that allow them to monitor nutrient availability in their rhizosphere and adjust their metabolism, growth patterns, and resource allocation accordingly. The coordinated regulation of nutrient uptake, assimilation, and remobilization involves complex transcriptional networks, post-translational modifications, and metabolic reprogramming. This Research Topic showcases how cutting-edge approaches, from genome-wide transcriptomics and metabolomics to functional characterization of transcription factors and field validation, are converging to elucidate these adaptive mechanisms across diverse plant species.

## Transcriptional regulation of nitrogen homeostasis in rice

A central theme emerging from this Research Topic is the pivotal role of transcription factors in orchestrating plant responses to nutrient stress. Du et al. demonstrate that the rice transcription factors OsNIGT2 and OsNIGT3, members of the GARP family, function as transcriptional repressors that fine-tune nitrogen acquisition under high-nitrogen conditions. Through comprehensive DNA affinity purification sequencing (DAP-seq) and functional validation, they reveal that OsNIGT2 and OsNIGT3 function as nitrate-induced regulators that downregulate key nitrate and ammonium transporters (OsNRTs, OsAMTs), thereby acting as a molecular “brake” on nitrogen acquisition under high-N supply to prevent excessive nitrogen uptake. Through CRISPR-generated mutants, multi-omics analyses, and field validation, the work establishes functional redundancy between the two transcription factors and shows that dual knockout enhances nitrogen accumulation, biomass, agronomic NUE and increased grain yield in field trials, underscoring their potential as genetic targets for crop improvement. However, it is important to note that OsNIGT2/3 act primarily under nitrate-rich conditions and show limited activity during low-nitrogen stress, which poses a key limitation for low-input agriculture where enhancing performance under N scarcity is critical.

This regulatory paradigm extends beyond nitrogen metabolism. The same NIGT family members have been implicated in coordinating nitrogen and phosphate signaling pathways, illustrating the deeply interconnected nature of nutrient-sensing networks. The work highlights how evolution has shaped multifunctional regulatory modules that allow plants to integrate information about multiple nutrient availability simultaneously, optimising resource allocation in complex soil environments.

## Cross-species insights: from model systems to orphan crops

While much of our mechanistic understanding derives from model species such as Arabidopsis and rice, Wang et al. expand this knowledge to cassava, a vital staple crop for millions globally yet one of the most understudied in terms of molecular physiology. Their comprehensive multi-omics investigation into the physiological, transcriptional, and metabolic responses of cassava seedlings under nitrogen (N) deficiency revealed dramatic metabolic reprogramming. Low nitrogen conditions triggered significant accumulation of flavonoids and other secondary metabolites, alongside reduced photosynthetic capacity and altered carbon-nitrogen partitioning. The identification of cassava-specific transcription factors regulating flavonoid biosynthesis under nitrogen stress provides important genetic and metabolic insights for future nitrogen-use-efficiency (NUE) breeding in this resilient but yield-limited crop.

This study shows how systems biology approaches, such as combining physiological measurements, genome-wide expression profiling, and untargeted metabolomics, can rapidly accelerate our understanding of nutrient stress responses in non-model species. This is particularly valuable given that many climate-resilient crops adapted to marginal soils remain poorly characterized at the molecular level.

## Phosphorus efficiency through novel regulatory pathways

Turning to phosphorus metabolism, Hussain et al. provide a compelling perspective on the ZmNF-YC1–ZmAPRG pathway in maize, demonstrating how Nuclear Factor Y transcription factors integrate phosphate stress signals to modulate not only phosphate homeostasis but also lipid metabolism and photosynthetic efficiency. Under phosphorus-deficient conditions, ZmNF-YC1 forms a heterotrimeric complex that activates ZmAPRG, triggering lipid remodeling where phospholipids are replaced by non-phosphorus lipids such as MGDG and DAG. This metabolic adjustment maintains membrane integrity while releasing phosphorus for more critical cellular functions. What makes this pathway particularly intriguing is its potential for multifunctional stress tolerance. Given that NF-Y transcription factors have been implicated in responses to drought, salinity, and temperature stresses, the authors speculate that manipulating the ZmNF-YC1–ZmAPRG axis could yield maize varieties with enhanced resilience to multiple environmental challenges. While this hypothesis awaits experimental validation, it represents an exciting frontier in the development of climate-smart crops.

Complementing this mechanistic work, Meng et al. conduct a genome-wide characterization of the GDPD gene family in foxtail millet, identifying 14 members with distinct expression patterns under phosphorus starvation. Through heterologous expression in Arabidopsis, they demonstrate that SiGDPD14 confers enhanced tolerance to low phosphorus stress, manifested through improved root growth and seed germination under phosphorus limitation. The enzymatic activity of GDPDs in hydrolyzing glycerophosphodiesters to release inorganic phosphate makes them attractive targets for bioengineering improved phosphorus acquisition and recycling.

## Nutrient interactions in wetland ecosystems: nitrogen and phosphorus dynamics

Beyond agricultural systems, understanding nutrient dynamics in natural and semi-natural ecosystems is crucial for water quality management and ecological restoration. Huang et al. investigate the interactions between nitrogen and phosphorus during flooding-induced decomposition of Cynodon dactylon in water-level fluctuation zones. Their study reveals that flooding significantly increases initial nitrogen and phosphorus levels in overlying water, particularly particulate nitrogen and particulate phosphorus, which profoundly affect plant decomposition rates and subsequent nutrient release dynamics.

Over a 60-day decomposition period, Cynodon dactylon lost 47.97% to 66.10% of its dry matter and released 43.58% to 54.48% of total nitrogen and 14.28% to 20.50% of total phosphorus. Notably, initial particulate nitrogen and particulate phosphorus promoted decomposition and phosphorus loss, while paradoxically inhibiting total nitrogen loss from the plant material. This suggests complex regulatory mechanisms governing nutrient release during litter decomposition. By day 60, no positive correlation was found between plant-released nutrients and those in overlying water, indicating that initial nutrient pulses from soil rather than gradual plant decomposition drive water quality changes in these fluctuating environments.

These findings have important implications for managing water-level fluctuation zones in reservoirs and wetlands, where the interaction between soil-derived nutrients and plant decomposition influences water purification processes. The study demonstrates that nitrogen and phosphorus released from flooded soils can support microbial aggregate formation, potentially enhancing denitrification and phosphorus removal—critical processes for maintaining water quality in these transitional ecosystems. While focused on wetland ecosystems, these insights into nutrient release dynamics, microbial-mediated transformations, and the timing of nutrient pulses from flooded soils have direct applications to rice paddies and other flooded agricultural systems, where managing water-nutrient interactions is crucial for optimising both crop productivity and environmental sustainability.

## Emerging themes and future directions

Several cross-cutting themes emerge from these studies that merit further exploration:

### Nutrient crosstalk and integration

The interconnection between nitrogen and phosphorus signaling pathways is no longer speculative but mechanistically demonstrated. NIGT family transcription factors serve as molecular integration points, capable of sensing multiple nutrient inputs and coordinating appropriate transcriptional responses. Future work should investigate how these regulatory nodes prioritize responses when plants face simultaneous deficiencies of multiple nutrients—a common scenario in agricultural soils.

### Root architecture and below-ground foraging

While the molecular mechanisms of nutrient sensing are becoming clearer, the relationship between these genetic networks and root system architecture remains underexplored in several articles. Enhanced root hair development, lateral root proliferation, and altered root-to-shoot ratios are hallmark responses to nutrient stress, yet the connections between transcriptional regulators like NIGT2/3 or NF-YC1 and developmental programs controlling root morphology require further investigation. Understanding these connections is essential for breeding varieties that can efficiently utilize nutrients in heterogeneous soil environments.

### Secondary metabolism as an adaptive response

The accumulation of flavonoids and other phenylpropanoid pathway metabolites under nitrogen deficiency, as documented in cassava and corroborated by work in other species, raises intriguing questions about the adaptive significance of this metabolic shift. Are these compounds serving protective roles against oxidative stress induced by nutrient limitation? Do they function in root exudation to modify rhizosphere chemistry? Unravelling the functional significance of secondary metabolite accumulation could reveal novel intervention points for enhancing stress tolerance.

### From genes to fields: bridging the laboratory-field divide

One of the strengths of the OsNIGT2/3 study is the demonstration of improved agronomic nitrogen use efficiency and yield in actual field conditions. Too often, promising laboratory findings fail to translate into real-world agricultural benefits. The field validation presented here serves as a model for how functional genomics research should be conducted, with clear pathways from molecular discovery to practical application. Similar field-based validation is needed for the phosphorus-related pathways and the cassava findings to assess their potential for contributing to sustainable intensification of agriculture.

### Toward sustainable agriculture

Global agriculture faces a profound challenge: producing more food on finite land while reducing reliance on synthetic fertilizers that contribute to greenhouse gas emissions, eutrophication of waterways, and soil degradation. Nitrogen fertilizers account for approximately 2% of global energy consumption and 1.2% of CO_2_ emissions, while phosphorus is a non-renewable resource with finite global reserves. Developing crop varieties with enhanced nutrient use efficiency is therefore not merely an agronomic optimization but an environmental and economic imperative.

The research compiled here provides a robust foundation for this endeavor. The identification of specific transcription factors and metabolic enzymes that can be targeted through either conventional breeding or genome editing offers tangible pathways forward. Importantly, the work demonstrates that nutrient efficiency is not a monolithic trait but involves coordinated regulation across multiple scales—from individual transporter proteins to whole-plant resource allocation strategies.

## Conclusion

This Research Topic illustrates how integrative approaches spanning molecular genetics, systems biology, and agronomy are transforming our understanding of plant nutrient stress responses. The convergence of mechanistic depth, exemplified by the characterization of NIGT transcription factors and NF-Y regulatory complexes, with translational relevance demonstrated through field trials and work in crop species, positions this research at the forefront of efforts to develop climate-resilient, resource-efficient agriculture.

As we look to the future, several challenges remain. We need to better understand how plants integrate signals from multiple nutrient deficiencies, how regulatory networks are modified during evolutionary adaptation to different soil types, and how laboratory discoveries can be efficiently translated into varieties that perform under the variable conditions of real-world agriculture. The work presented here provides an essential foundation for addressing these questions and moves us closer to a future where crops can produce abundant yields while minimizing environmental impact.

The path forward requires continued collaboration across disciplines, from molecular biology and biochemistry to ecology and agricultural sciences. Yet collaboration alone is insufficient, we must also embrace a more ambitious vision. The genetic targets identified here, from OsNIGT2/3 in rice to ZmNF-YC1 in maize to SiGDPD14 in foxtail millet, represent not merely individual breeding opportunities but components of a larger regulatory architecture that coordinates plant responses to the complex, multi-nutrient reality of agricultural soils. The critical next step is to move beyond optimizing plants for single-nutrient deficiencies toward engineering varieties that can dynamically balance nitrogen and phosphorus acquisition in response to fluctuating soil conditions. This will require integrating the molecular insights presented here with field-based phenotyping, predictive modeling of genotype-by-environment interactions, and participatory breeding approaches that engage farmers in variety selection. Can we meet this challenge within the next decade? The convergence of CRISPR-based precision breeding, high-throughput phenotyping, and systems biology approaches suggests we can, but only if we commit to the long-term, interdisciplinary partnerships necessary to translate molecular discoveries into agricultural impact.

